# High BLM Expression Predicts Poor Clinical Outcome and Contributes to Malignant Progression in Human Cholangiocarcinoma

**DOI:** 10.3389/fonc.2021.633899

**Published:** 2021-03-22

**Authors:** Xiaolong Du, Chen Zhang, Chuanzheng Yin, Wenjie Wang, Xueke Yan, Dawei Xie, Xichuan Zheng, Qichang Zheng, Min Li, Zifang Song

**Affiliations:** ^1^Department of Hepatobiliary Surgery, Union Hospital, Tongji Medical College, Huazhong University of Science and Technology, Wuhan, China; ^2^State Key Laboratory of Molecular Oncology, National Cancer Center/National Clinical Research Center for Cancer/Cancer Hospital, Chinese Academy of Medical Sciences and Peking Union Medical College, Beijing, China

**Keywords:** cholangiocarcinoma, prognosis, progression, co-expression network analysis, bioinformatics

## Abstract

Molecular mechanisms underlying the tumorigenesis of a highly malignant cancer, cholangiocarcinoma (CCA), are still obscure. In our study, the CCA expression profile data were acquired from The Cancer Genome Atlas (TCGA) database, and differentially expressed genes (DEGs) in the TCGA-Cholangiocarcinoma (TCGA-CHOL) data set were utilized to construct a co-expression network *via* weighted gene co-expression network analysis (WGCNA). The blue gene module associated with the histopathologic grade of CCA was screened. Then, five candidate hub genes were screened by combining the co-expression network with protein–protein interaction (PPI) network. After progression and survival analyses, bloom syndrome helicase (BLM) was ultimately identified as a real hub gene. Moreover, the receiver operating characteristic (ROC) curve analysis suggested that BLM had a favorable diagnostic and predictive recurrence value for CCA. The gene set enrichment analysis (GSEA) results for a single hub gene revealed the importance of cell cycle-related pathways in the CCA progression and prognosis. Furthermore, we detected the BLM expression *in vitro*, and the results demonstrated that the expression level of BLM was much higher in the CCA tissues and cells relative to adjacent non-tumor samples and normal bile duct epithelial cells. Additionally, after further silencing the BLM expression by small interfering RNA (siRNA), the proliferation and migration ability of CCA cells were all inhibited, and the cell cycle was arrested. Altogether, a real hub gene (BLM) and cell cycle-related pathways were identified in the present study, and the gene BLM may be involved in the CCA progression and could act as a reliable biomarker for potential diagnosis and prognostic evaluation.

## Introduction

Cholangiocarcinoma (CCA) is one of the most frequent primary biliary duct malignancies and accounts for 3% of all gastrointestinal neoplasias (1, 2). Since CCA possesses the characteristics of high malignancy, insidious onset, and rapid progression, the prognosis of patients with CCA is often poor (3, 4). Large-scale clinical research revealed that the 5-year survival rates were 5 and 17% for intrahepatic CCA (iCCA) and extrahepatic CCA (eCCA), respectively, for the cases diagnosed between 2000 and 2007 in Europe (5). In the USA alone, CCA accounts for approximately 5,000 deaths per year (6). Moreover, since its early symptoms are not obvious and CCA lacks a highly efficient disease screening biomarker, many patients have frequently lost the chance of radical surgery upon the first diagnosis (7). Furthermore, as the pathogenesis of CCA is rather complicated, there is still no satisfying targeted treatment option available for CCA in the age of individualized medicine (8). Therefore, there is an urgent need to explore the molecular pathogenesis of CCA and reveal the biomarkers closely related to the diagnosis, occurrence, progression, and prognosis of CCA.

Due to advances in sequencing and microarray technologies, bioinformatics has begun to play an increasingly important role in various fields of life sciences (9, 10). The development of large-scale “omics” research methods such as genomics, proteomics, and transcriptomics has brought about a vast amount of biological information data (11). Gene co-expression networks can describe and cluster multiple genes sharing high-expression correlations in microarray data or high-throughput sequencing data and ultimately be used to establish key regulatory genes (12). A weighted gene co-expression network analysis (WGCNA), as one of the most representative analytical methods of a gene network, has provided significant biological information for the research on multiple species such as humans, mice, and yeast (13, 14). In this study, a WGCNA network was constructed, and genes with similar expression patterns were incorporated into the identical modules. By relating the results of the module to the corresponding clinical data, the modules that were mostly associated with the CCA progression were found. Finally, after a range of screening and validation tests, we identified a bloom syndrome helicase (BLM) gene that could indeed promote the tumor progression and predict the prognosis of CCA.

## Materials and Methods

### Data Sets and Study Design

RNA-sequencing (RNA-seq) data (Illumina RNA Seq V2, Illumina, San Diego, CA) and the corresponding clinical data on CCA samples were downloaded from The Cancer Genome Atlas (TCGA) database (http://cancergenome.nih.gov/). This CHOL data set included 36 CCA samples and 9 corresponding adjacent non-cancerous samples, and its clinical data were composed of tumor histologic grade, pathological stage, and numerous follow-up information. The messenger RNA (mRNA) expression profile and clinical data were employed to search for differentially expressed genes (DEGs) and construct co-expression networks. The microarray data sets GSE76297 and GSE132305 were acquired from the gene expression omnibus (GEO) database of the National Center for Biotechnology Information (NCBI) database (https://www.ncbi.nlm.nih.gov/). These two data sets were generated on the platforms of the Affymetrix Human Transcriptome Array 2.0 and Affymetrix Human Genome U219 Array, respectively. The data set GSE76297 contained 91 iCCA tissue and 92 non-neoplastic samples, and the data set GSE132305 included 182 eCCA samples and 38 corresponding adjacent non-cancerous samples. These two data sets were used to further validate our candidate hub genes.

### Differentially Expressed Gene Screening and Principal Component Analysis

The “TCGAbiolinks” R package (15) was used to identify the DEGs between adjacent normal tissues and CCA samples in the CHOL data set. Significance analysis with a false discovery rate (FDR) < 0.01 and |log_2_ fold change (FC)| ≥ 1 was adopted to choose the genes for a network construction. To explore gene expression patterns between the CCA samples and adjacent tissues, the top 500 DEGs were included in the principal component analysis (PCA) utilizing the R package “pca3d” (https://cran.r-project.org/web/packages/pca3d/). The first three principal components were utilized and plotted to show the expression pattern of the two groups.

### Weighted Gene Co-expression Network Analysis

R package of “WGCNA” (16) was utilized to build a co-expression network for the qualified expression data profiles of filtered DEGs with the available clinical data. First, we retained the RNA-seq data of the filtered CCA tissues if they proved to be good samples. Second, outlier samples were culled after a sample clustering *via* correlation analysis. Third, based on a Pearson correlation analysis, a matrix of similarity for all pairs of kept genes was constructed. Then, appropriate soft-thresholding was chosen to achieve a scale-free co-expression network. Next, the adjacency matrix was converted to a topological overlap matrix (TOM). In line with the TOM-based dissimilarity method, genes were assigned to the different modules. Here, we set the soft-thresholding power to 9 (scale-free *R*^2^ = 0.913), the cut height to 0.25, and the minimal module size to 30 to identify key modules. The module most relevant to clinical traits was chosen to screen co-expression hub genes.

### Function Enrichment Analyses

We performed gene ontology (GO) and Kyoto Encyclopedia of Genes and Genomes (KEGG) enrichment analyses utilizing the R package “clusterprofiler” (17). GO terms or KEGG pathways with *p* < 0.05 were regarded as statistically significant and visualized by R package “GOplot” (18).

### Candidate Hub Genes Identification

In this research, a module of interest was determined, and co-expression hub genes were specified as high module connectivity (module membership > 0.8) and high clinical trait significance (gene significance > 0.4). Moreover, the gene list of this key module was submitted to the STRING database to build a protein–protein interaction (PPI) network (19), which was then visualized by Cytoscape software (20). PPI network hub genes were defined as the top 20 nodes ranked by degree using the plugin cytoHubba. Hub genes shared by the 2 networks were selected as the candidates for further validation and analysis.

### Validation and Survival Analysis of Candidate Hub Genes

To screen real hub genes, we used R package “ggstatsplot” to explore the candidate gene expression pattern between CCA and adjacent normal tissue in the TCGA-CHOL data set, GSE76297, and GSE132305; the candidate genes that were correlated with a clinical feature (histological grade) were analyzed by using the tumor samples from the TCGA-CHOL data set. Survival analysis was also performed for the candidate genes in gene expression profiling interactive analysis (GEPIA), an online database (http://gepia.cancer-pku.cn/index.html), and validated in the data set GSE107943 by using online web server OSchol (http://bioinfo.henu.edu.cn/CHOL/CHO-L_GSE107943.jsp). Independent sample *t*-tests were used as appropriate, and *p* < 0.05 was considered as statistically significant.

### Methylation Analyses and Efficacy Evaluation of Real Hub Genes

An online tool DiseaseMeth 2.0 (21) contains a mass of methylome data and annotations on the DNA methylation status in multiple diseases. We exploited this web tool to compare the methylation level of the real hub gene between the CCA and adjacent normal tissues. Additionally, the association between the BLM expression and DNA methylation status was explored *via* the web tool MEXPRESS (22), which can integrate and visualize gene expression, DNA methylation, and clinical information from the TCGA database. The receiver operating characteristic (ROC) curves were plotted, and the area under the curve (AUC) was calculated with the “pROC” R package (23) to assess the capability of BLM gene of distinguishing tumor and normal samples as well as recurrent and non-recurrent CCA.

### Gene Set Enrichment Analysis

Based on the median expression value of the real hub gene, CCA samples of the TCGA-CHOL data set were divided into low- or high-expression groups. To explore potential functions of the real hub gene, gene set enrichment analysis (GSEA) was performed to detect which KEGG pathways were enriched (24). Terms with FDR < 0.05 were visualized by R package “ggplot2” (https://cloud.r-project.org/web/packages/ggplot2/).

### Cell Culture

Human CCA cell lines (QBC939 and RBE) and human biliary epithelial cell line (HiBEC) were obtained from Sciencell (Carlsbad, USA) and the Cell Bank of the Chinese Academy of Sciences (Shanghai, China) and maintained in Roswell Park Memorial Institute (RPMI) 1,640 medium (Gibco, NY, USA) containing 10% fetal bovine serum (FBS) (ScienCell, San Diego, CA, USA) and 100 U/ml penicillin-streptomycin (Beyotime, Shanghai, China) at 37°C in a humidified incubator supplied with 5% CO2.

### Transfection

Small interfering RNA (SiRNA) silencing was attained by transient transfection with Lipo3000 (Invitrogen, Waltham, MA, USA). BLM siRNAs (si-RNA1 and si-RNA2) were purchased from RiboBio (RiboBio, Guangzhou, China), with scramble RNA serving as a negative control (scramble). The sequences for si-RNA1 and si-RNA2 were 5′-GGATGTTCTTAGCACATCA-3′ and GACTCAGAATGGTTAAGCA, respectively. The scramble sequence was 5′- TTCTCCGAACGTGTCACGTdTdT-3′. The siRNA knockdown efficiency was examined by qRT-PCR and western blotting assays.

### CCK8 Assay

Cells transfected with si-BLM or scramble were seeded into 96-well-plates at 4 × 10^3^ cells per well and cultured for 0–3 days. Then, 10 μl of CCK8 solution (Dojindo, Kumamoto, Japan) was added to the fresh culture medium, and the cells were incubated at 37°C for 1.5 h. Afterward, the absorbance at 450 nm was determined by using a microplate reader.

### Wound-Healing Assay

For the wound-healing assay, transfected cells were inoculated into 6-well-plates. After the cell attachment, a sterile plastic microtube head was used to generate scratch wounds. Then, the cells were washed twice with PBS, and initial wounds were recorded by a microscope. After incubation for 36 h at 37°C, the current wound space was captured. The quantity of wound closure was defined as the mean percentage of the distance of cell migration compared with the initial wound width.

### Transwell Assay

Cell migration ability was further evaluated *via* transwell assays. Briefly, transfected cells (3 × 10^4^) were plated into 8.0-μm pore polycarbonate membrane chambers (Corning, NY, USA) with 200 μl of serum-free medium, and then the chambers were placed in 24-well-plates containing 600 μl of complete medium. After 24 h, the cells migrating to the lower surface were fixed with a formaldehyde solution and stained with 0.1% crystal violet solution. The number of migrated cells was counted in five randomly selected visual fields of each membrane with a microscope.

### Quantitative Real-Time PCR Analysis

Total RNA from the cultured cells was extracted by using TRIzol (Invitrogen, Carlsbad, CA, USA), and then it was reverse transcribed into cDNA *via* PrimeScript RT Master Mix (Takara, Dalian, China). Subsequently, quantitative real-time PCR (RT-qPCR) was conducted by utilizing the TAKARA PCR Kit, run on a StepOnePlus Real-Time PCR system (Applied Biosystems, CA, USA). β-actin was chosen as an internal reference. The relative expression abundance of genes was assessed by using the 2^−ΔΔCT^ approach (25). Primers were provided as follows: β-actin, forward 5′-TGCTATGTTGCCCTAGACTTCG-3′ and reverse 5′-GTTGGCATAGAGGTCTTT ACGG-3′; and BLM, forward 5′- CAGACTCCGAAGGAAGTTGTATG' and reverse 5′- TTTGGGGTGGTGTAACAAATGAT-3′.

### Western Blot Analysis

For the total protein extraction, the treated cells were lysed by RIPA buffer (Beyotime, Shanghai, China) with protease inhibitors (Servicebio, Wuhan, China). Then, the same amount of whole-cell lysates was loaded on the sodium dodecyl sulfate-polyacrylamide gel electrophoresis (SDS-PAGE) and then transferred onto PVDF membranes. After 1 h of blocking, proteins were incubated with anti-β-actin antibodies (abs132001, Absin, Shanghai, China), anti-GAPDH antibodies (abs132004, Absin, Shanghai, China), and anti-BLM antibodies (abs122169, Absin, Shanghai, China) and subsequently with appropriate secondary antibodies (Servicebio Technology, Wuhan, China). Protein bands were detected and quantified *via* ImageLab (Bio-Rad, Hercules, CA, USA).

### Immunohistochemistry Staining

A tissue microarray (TMA) containing 27 CCA specimens and 9 paracancerous samples was procured from Shanghai Outdo Biotech (Shanghai, China). In short, paraffin sections were first deparaffinized, antigen retrieval was conducted in citrate buffer (pH 6.0), and endogenous peroxidase activity was blocked in 0.3% H_2_O_2_. The slides were continuously incubated with the indicated primary and secondary antibodies until visualization with peroxidase and 3,30-diaminobenzidine tetrahydrochloride. Immunohistochemical staining was scored semiquantitatively according to the percentage and intensity of positively stained cells as follows: 0: <5% positive cells; 1: from 5 to 24% positive cells; 2: from 25 to 49% positive cells; 3: from 50 to 74% positive cells; and 4: more than 75% positive cells. The intensity was scored as 0 for the absence of staining, 1 for weak, 2 for moderate, and 3 for strong staining. Staining index score = intensity × positive rate (absent, 0–1; mild, 2–4; moderate, 5–8; and strong, 12). A staining index score ≥8 represented a high expression, while scores <8 represented a low expression. Immunohistochemical slices were observed by utilizing an Olympus BX microscope.

### Flow Cytometry

The treated cells were washed with pre-cooled PBS twice and fixed in 70% pre-cooled ethanol overnight at 4°C and washed again with PBS twice. The cells were resuspended in 1 ml PI/Triton X-100 staining solution containing 0.2 mg RNase A for 15 min at 37°C and then analyzed *via* flow cytometry. Cells were assayed at each cell cycle.

### Statistical Analysis

Each cell experiment was carried out thrice. All measurement data were presented as the mean ± SEM. Statistical data analysis was conducted by a one-way ANOVA or unpaired two-tailed Student's *t*-test, as appropriate. Values of *p* < 0.05 were deemed to indicate statistical significance.

## Results

### Screening of DEGs

**Figure 1:**
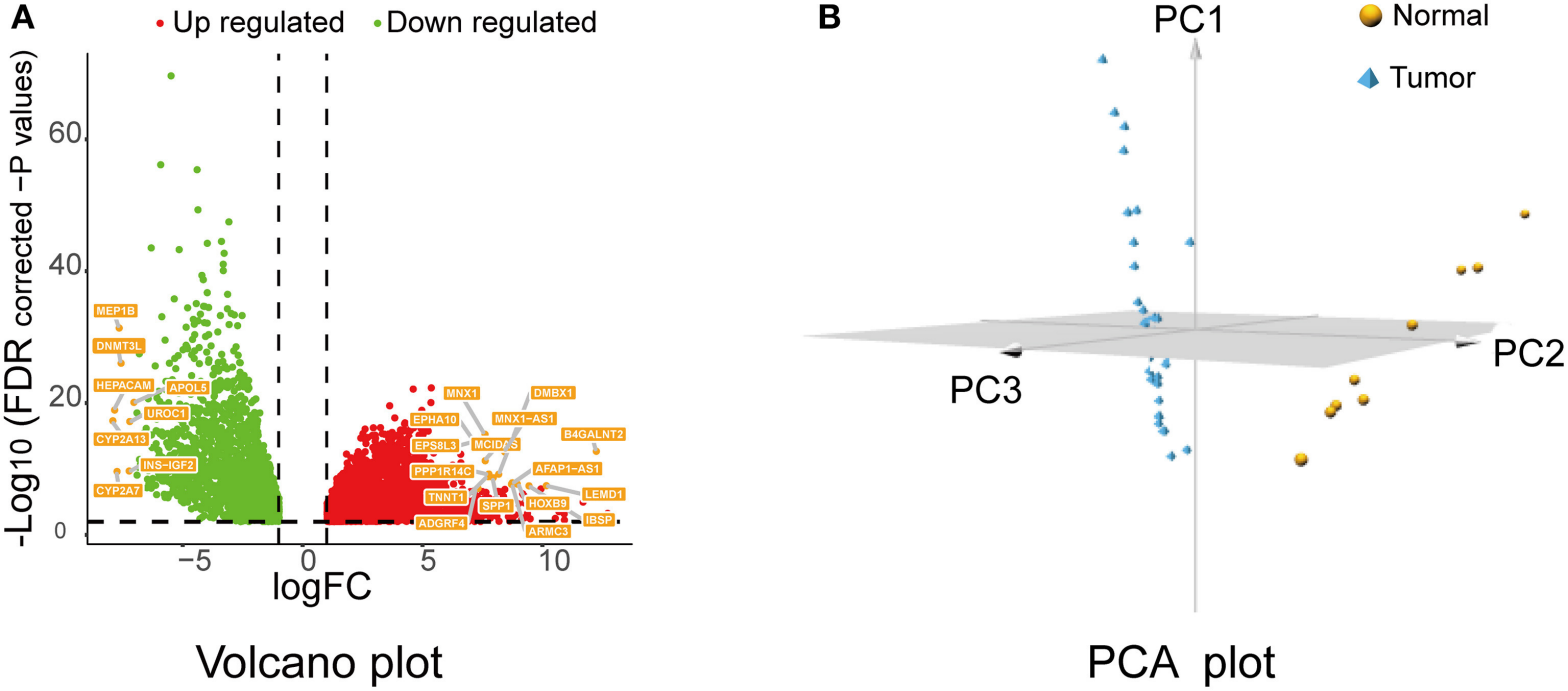
Volcano plot and PCA of DEGs. **(A)** Volcano plot of the DEGs between cholangiocarcinoma (CCA) tissues and normal controls of The Cancer Genome Atlas (TCGA) CHOL data set. Red means upregulated DEGs, and green indicates downregulated DEGs. **(B)** PCA plot for the TCGA-CHOL data set based on top 500 DEGs. PCA, principal component analysis; DEGs, differentially expressed genes.

After data preprocessing, data normalization, data filtering, and quality assessment by “TCGAbiolinks,” 6,219 DEGs were identified (4,087 upregulated and 2,132 downregulated) with the following threshold: FDR < 0.01 and |logFC| ≥1. Then, the screened genes were utilized for a follow-up analysis. The volcano plot for all DEGs is displayed in Figure 1A; the PCA of the top 500 DEGs is shown in Figure 1B.

### WGCNA and Key Module Identification

**Figure 2:**
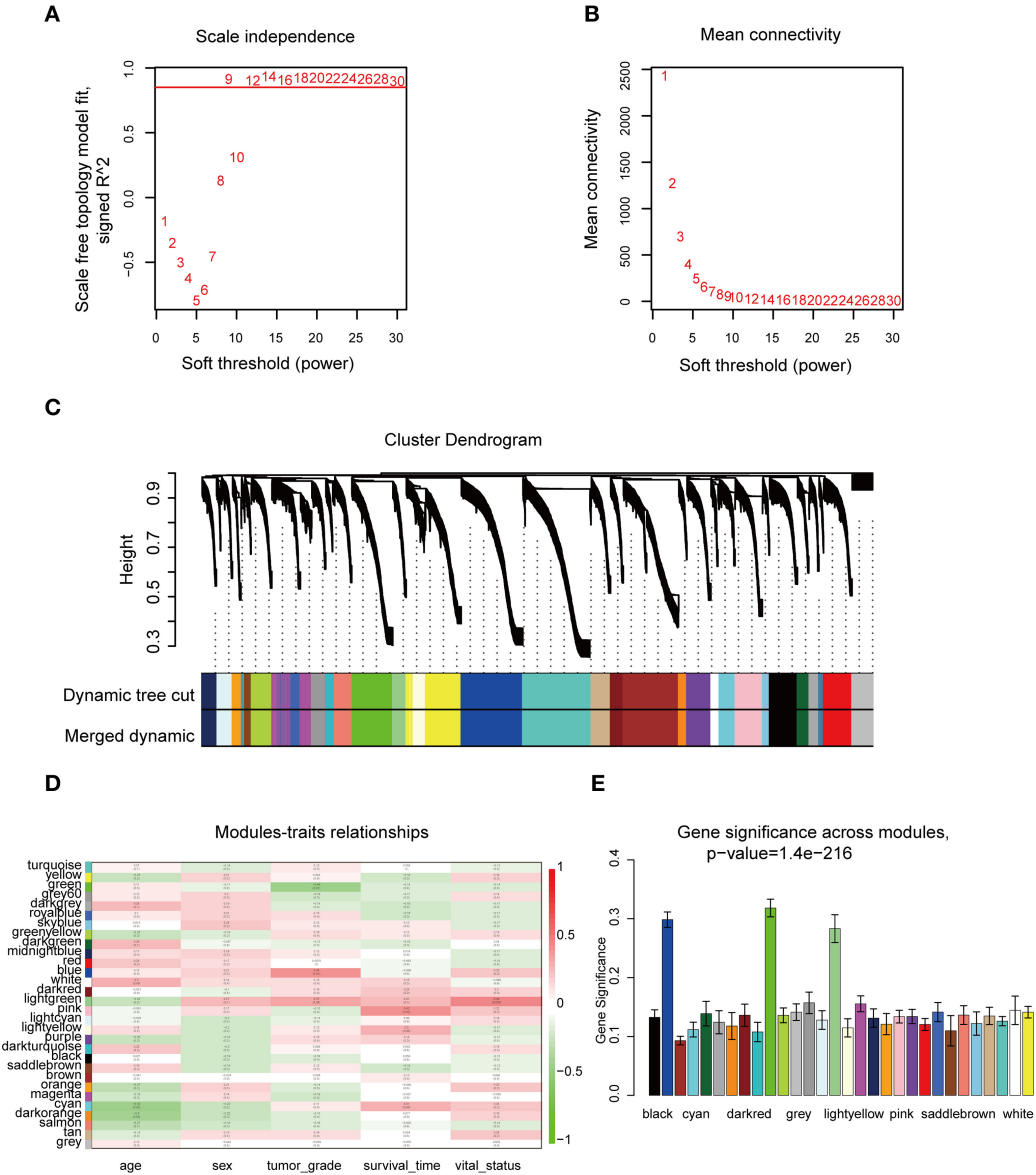
WGCNA of DEGs in CCA samples. **(A)** Examination of the scale-free fit index for distinct soft-thresholding powers (β). **(B)** Examination of the mean connectivity for distinct soft-thresholding powers. **(C)** Clustering dendrogram of filtered DEGs due to a dissimilarity measure. **(D)** Heatmap showing the connection between module eigengenes and five clinical traits of CCA. **(E)** Distribution of mean gene significance and errors in the modules correlated with tumor grade of CCA. WGCNA, weighted gene co-expression network analysis.

**Figure 3:**
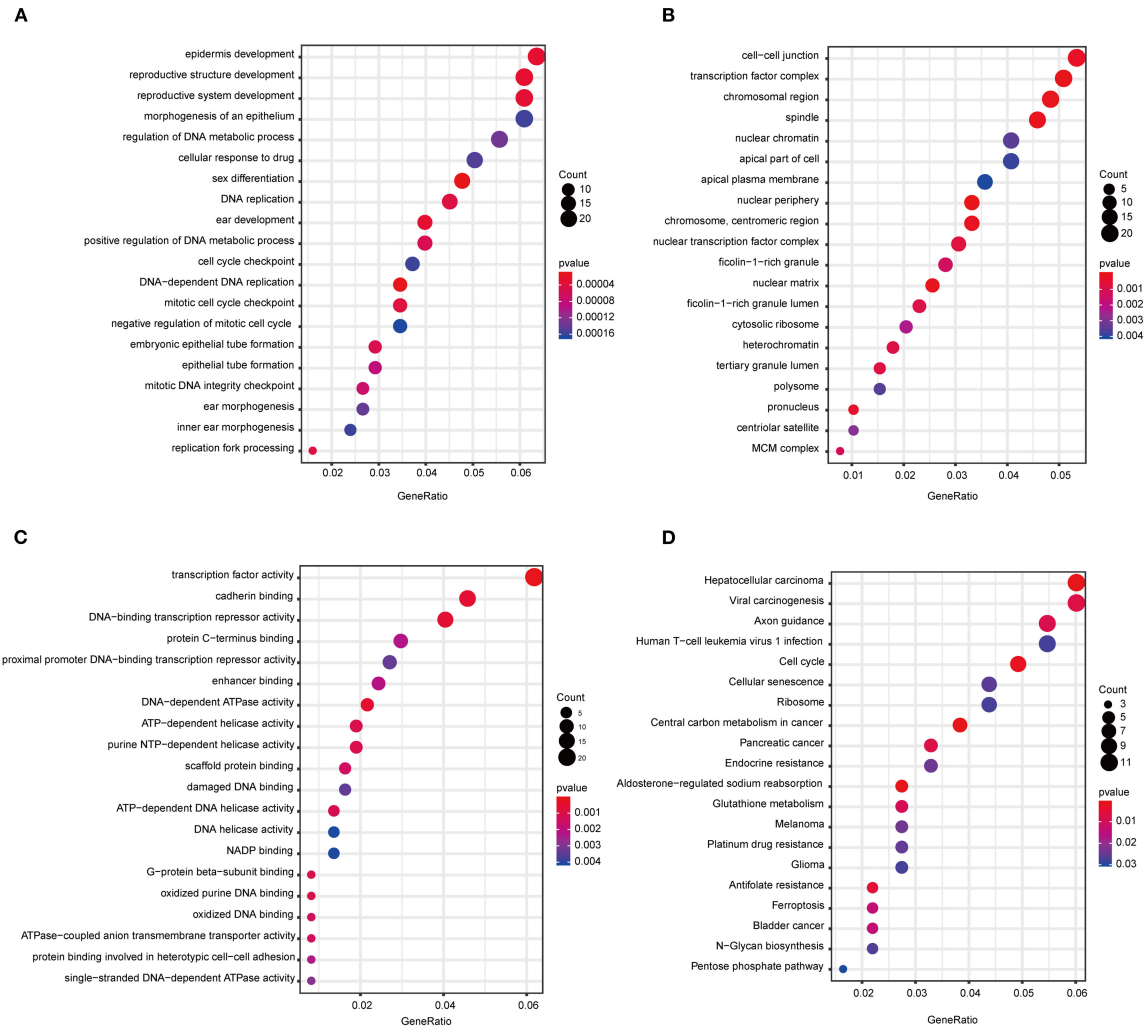
Functional annotation of all the blue module genes. **(A)** GO terms for biological process. **(B)** GO terms for cellular component. **(C)** GO terms for molecular function. **(D)** KEGG enrichment pathway for genes in the blue module. GO, gene ontology; KEGG, Kyoto Encyclopedia of Genes and Genomes.

The “WGCNA” R package was utilized to build a co-expression network, and DEGs with similar expression relationships were assigned into the same modules by average linkage clustering. Here, we selected the power of β = 9 (scale-free *R*^2^ = 0.913) to assure a scale-free network (Figures 2A,B), then 29 modules were determined (Figure 2C) in the subsequent experiments. The module that was most significantly correlated with a tumor grade indicated a great value in predicting the CCA progression, and the ME of the blue module indicated its high correlation with the CCA progression (Figure 2D). Moreover, we also found that the module significance (MS) of the blue module was the highest among all the modules that were positively correlated with disease progression (Figure 2E). Afterward, GO and KEGG analyses were performed to uncover the potential role of the members within the blue module. The most significant enrichment results are illustrated in Figures 3A–D. The biological function analysis suggested that blue module genes mainly participated in the cell cycle regulation and pathways in multiple cancers.

### Identification of Hub Genes

**Figure 4:**
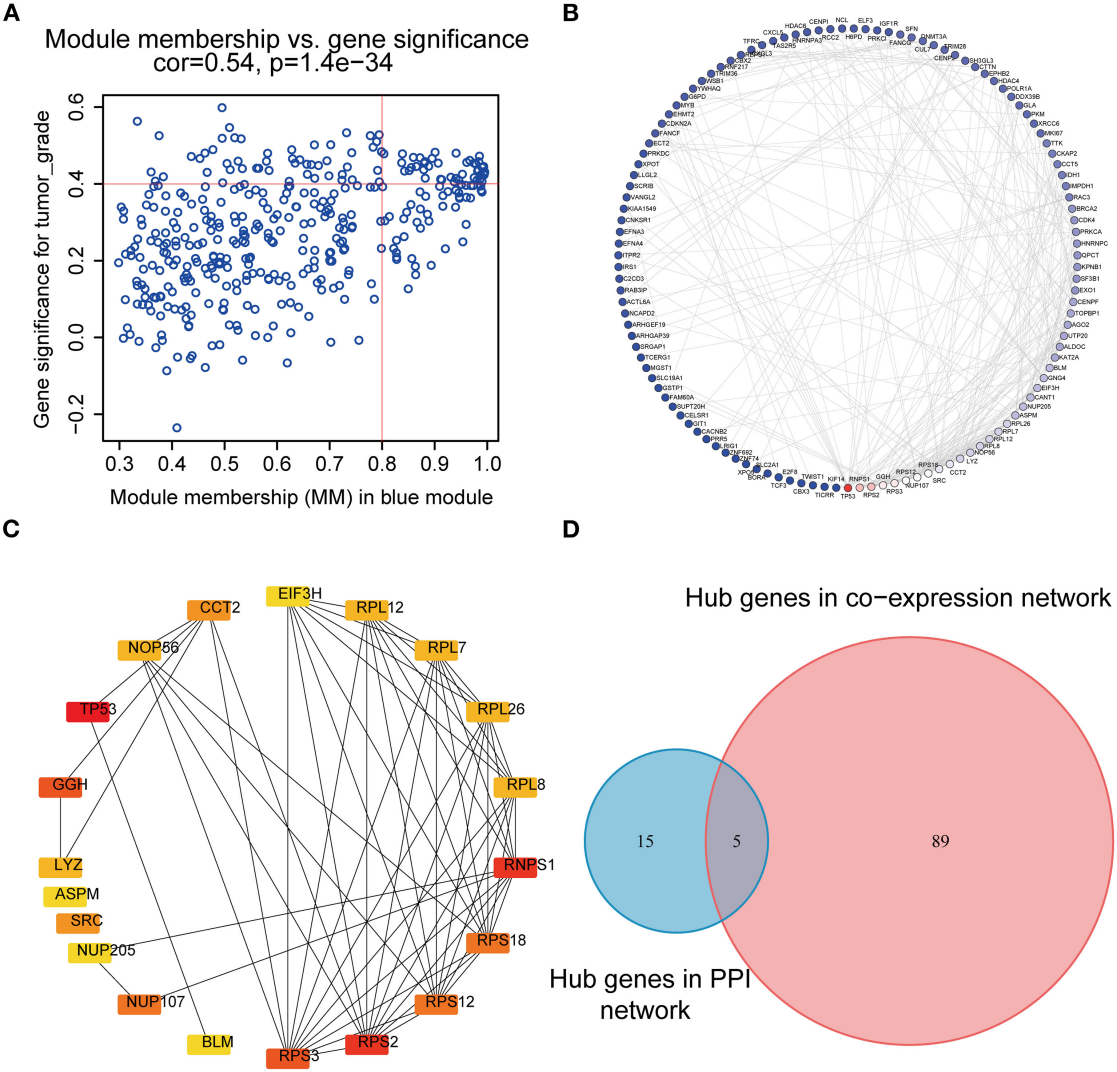
PPI network and hub gene detection. **(A)** Scatter plot of module eigengenes in the blue module. **(B)** PPI network for blue module gene. The color intensity of each node was proportional to the connectivity degree in the PPI network (highest connectivity degree in red and lowest connectivity degree in blue). **(C)** Top 20 hub nodes screened by the cytoHubba plugin of Cytoscape software. **(D)** Identification of candidate genes in the PPI network and co-expression network. PPI, Protein–protein interaction.

According to the scheme mentioned above, a total of 94 genes highly connected to the key module were selected as co-expression network hub genes in the blue module (Figure 4A). Additionally, we also built a PPI network for all blue module genes, which was composed of 123 nodes and 271 edges (Figure 4B). Then, using the cytoHubba plugin, the top 20 nodes associated with over 8 nodes were identified as hub genes in the PPI network (Figure 4C). Eventually, five common network hub genes—BLM, GGH, RPS3, NUP107, and CCT2—were identified as potential candidates for further validation and analysis (Figure 4D).

### Validation of Real Hub Gene

**Figure 5:**
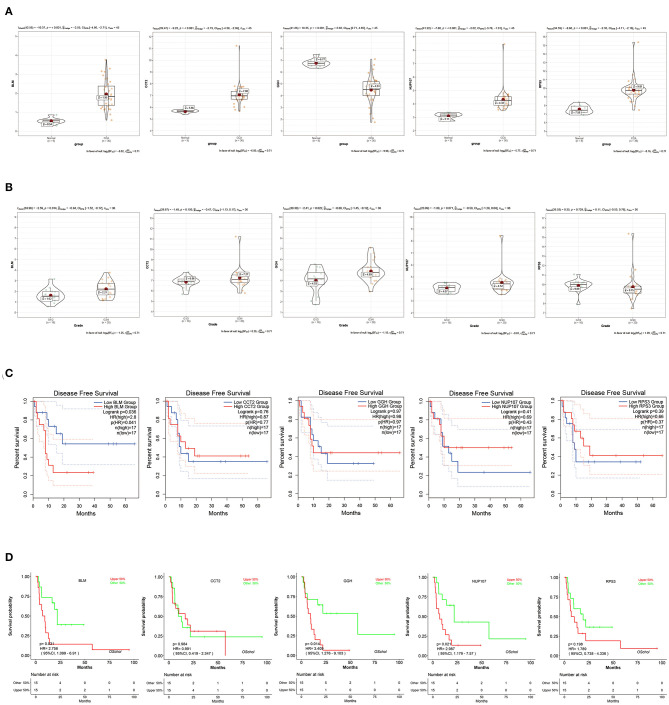
Validation of candidate hub genes in the TCGA-CHOL data set. **(A)** bloom syndrome helicase (BLM), CCT2, GGH, NUP107, and RPS3 gene expression contrast between CCA and non-tumorous adjacent samples. **(B)** Expression of BLM, CCT2, GGH, NUP107, and RPS3 in CCA samples with different pathological grades. **(C)** Association between BLM, CCT2, GGH, NUP107, and RPS3 expression and disease-free survival time in the TCGA-CHOL data set. The red line indicates samples with high gene expression (above the median value), and the blue line designates the samples with lowly expressed genes (below the median value). **(D)** Association between BLM, CCT2, GGH, NUP107, and RPS3 expression and disease-free survival time in the data set GSE107943. The red line indicates samples with high gene expression (above the median value), and the green line designates the samples with lowly expressed genes (below the median value).

**Figure 6:**
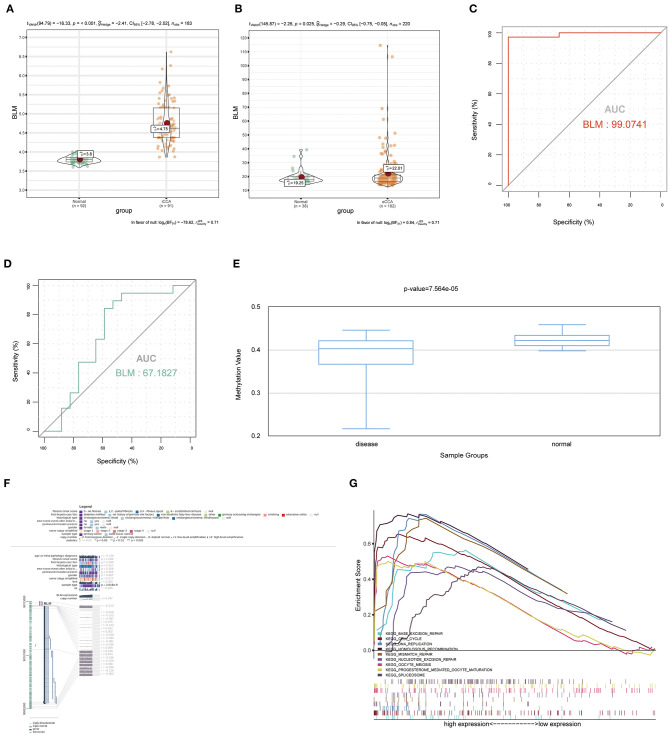
The expression of BLM in the external data sets of GSE76297 and GSE132305, the methylation level of BLM, the value of BLM in the diagnosis and prediction of recurrence, and gene set enrichment analysis (GSEA). **(A)** BLM gene expression differences between iCCA and adjacent non-tumor tissues of the iCCA data set GSE76297. **(B)** BLM gene expression differences between eCCA and adjacent non-tumor tissues of the eCCA data set GSE132305. ROC curves and AUC statistics were used to assess the capacity of BLM for CCA diagnosis **(C)** and to distinguish recurrent and non-recurrent CCA **(D)**. **(E)** The methylation levels of BLM in CCA and paracarcinoma tissue were examined by using DiseaseMeth 2.0. **(F)** The methylation sites of BLM and its associations with gene expression were visualized by using MEXPRESS. **(G)** GSEA using the TCGA-CHOL data set and statistically 9 significant functional gene sets enriched in CCA samples with high BLM expression were listed. iCCA, intrahepatic cholangiocarcinoma; eCCA, extrahepatic cholangiocarcinoma; ROC, receiver operating characteristic; AUC, area under the curve.

To verify the diagnostic value of the candidate genes, first, we examined the expression relationship of the candidate genes between CCA and normal controls using the TCGA-CHOL data set. It showed that the expression of candidate genes was upregulated in the CCA sample, except for the GGH gene (Figure 5A). Then, among the CCA samples in the TCGA-CHOL data set, we explored their correlations with the pathological grade of clinical features. The results suggested that four candidate genes were significantly higher in the low-differentiation tumor samples, indicating a role in the CCA progression (Figure 5B). To further examine the prognostic value of candidate genes in CCA, we conducted a subgroup (high- and low-expression group) survival analysis of these 5 genes in the TCGA-CHOL data set, and their prognostic value was validated in the data set GSE107943 by using the online tool OSchol (26). Kaplan–Meier (KM) survival curves indicated that only higher BLM expression was correlated significantly with worse disease-free survival (Figures 5C,D). Furthermore, the expression pattern of BLM in both the eCCA data set GSE132305 and the iCCA data set GSE76297 suggested that the expression of BLM in tumor tissue was enhanced compared with paracancerous samples (Figures 6A,B). ROC curves were then generated to assess the capacity of BLM to differentiate recurrent and non-recurrent CCA as well as tumor and non-tumor tissues (Figures 6C,D). The results above identified BLM as the real hub gene most closely related to the progression of CCA and as having an essential value in potential diagnosis and prognosis assessment of the disease.

### Relationship Between Methylation and Expression of the Real Hub Gene

We examined the relationship between the real hub gene's expression level and its methylation status to illuminate the potential cause of aberrant BLM expression in CCA. With the help of DiseaseMeth version 2.0, it revealed that the mean methylation level of BLM was significantly lower in CCA than in paracancerous normal tissue (Figure 6E). Additionally, we discovered that many methylation sites in the DNA sequence of gene BLM had a negative correlation with BLM expression using MEXPRESS (Figure 6F).

### GSEA of Real Hub Gene

To determine the potential function of BLM in CCA, GSEA was performed to seek significant pathways enriched in the sample group with high BLM expression. Nine gene sets, “BASE EXCISION REPAIR,” “CELL CYCLE,” “DNA REPLICATION,” “HOMOLOGOUS RECOMBINATION,” “MISMATCH REPAIR,” “OOCYTE MEIOSIS,” “PROGESTERONE MEDIATED OOCYTE MATURATION,” “SPLICEOSOME,” and “NUCLEOTIDE EXCISION REPAIR” were enriched (FDR < 0.05; Figure 6G). Overall, these gene sets were tightly associated with cell proliferation.

### BLM Expression Level Is Upregulated in CCA Cell Lines and CCA Tissue Samples

**Figure 7:**
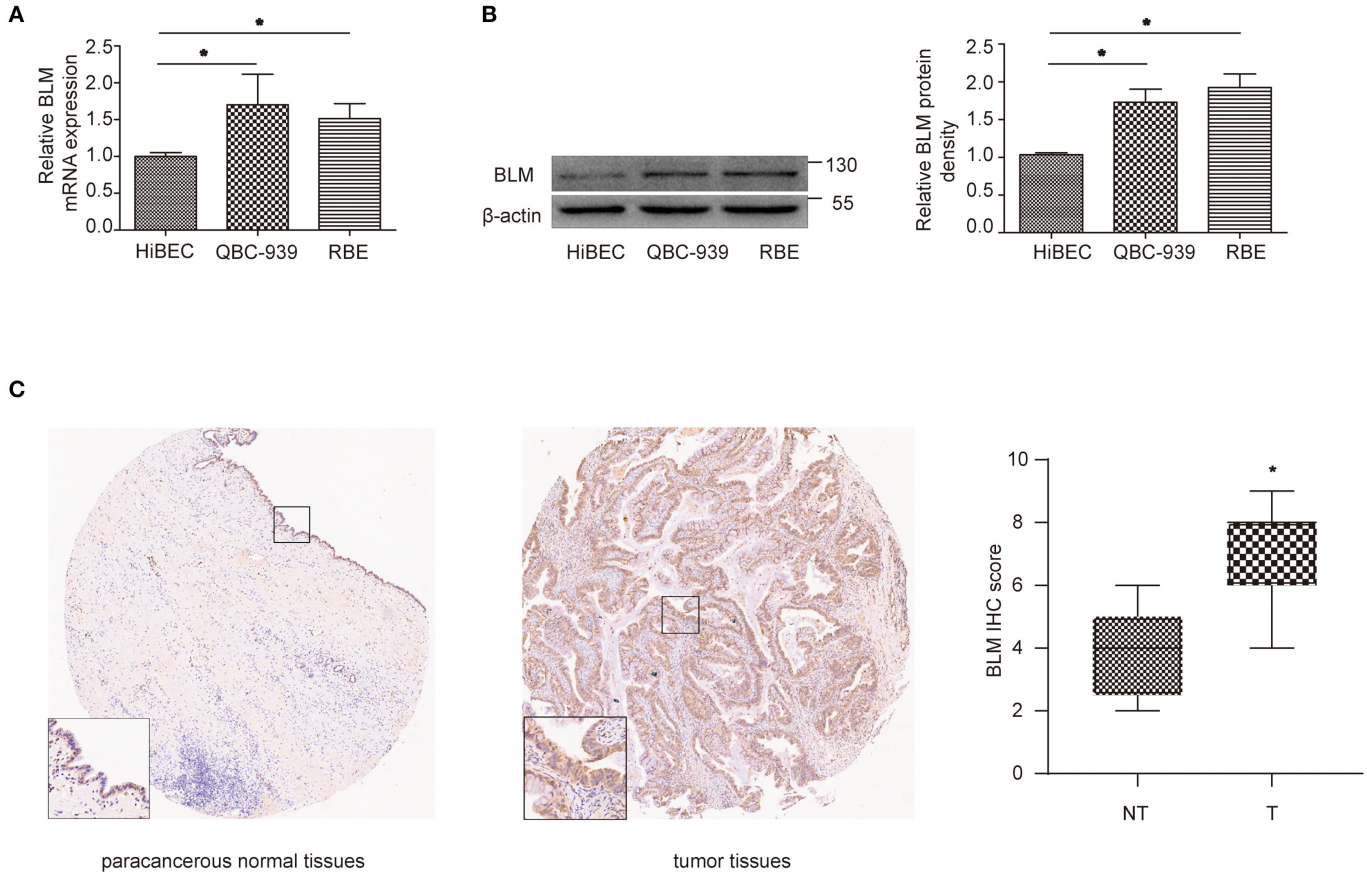
BLM expression was increased in CCA. **(A,B)** qRT-PCR **(A)** and western blot **(B)** analysis of BLM expression in human biliary epithelial cell lines (HiBECs), QBC-939 cells, and RBE cells. **(C)** Representative BLM immunohistochemistry (IHC) staining and score for NT and T samples. T, tumoral tissue; NT, corresponding adjacent non-tumoral tissues. Data are represented as the mean ± SEM of 3 independent experiments. *p < 0.05 vs. the control group.

Through the previous analysis, BLM was identified as the only real hub gene. We further investigated its expression level in different cell lines. The experiment results indicated that cancer cells (QBC939 and RBE) expressed higher levels of BLM than normal cells (HiBEC) at both the mRNA and protein levels (Figures 7A,B). We then conducted immunohistochemistry (IHC) assay to assess BLM expression utilizing a CCA TMA (including 27 CCA tissues and 9 paracancerous samples). As presented in Figure 7C, expression levels of BLM showed higher levels in CCA tissues compared with paracancerous samples (staining index: non-cancer = 3.78 ± 0.49; cancer = 6.78 ± 0.29).

### BLM Knockdown Inhibits the Proliferation and Migration of CCA Cells *in vitro* and Induced G2-Phase Arrest

**Figure 8:**
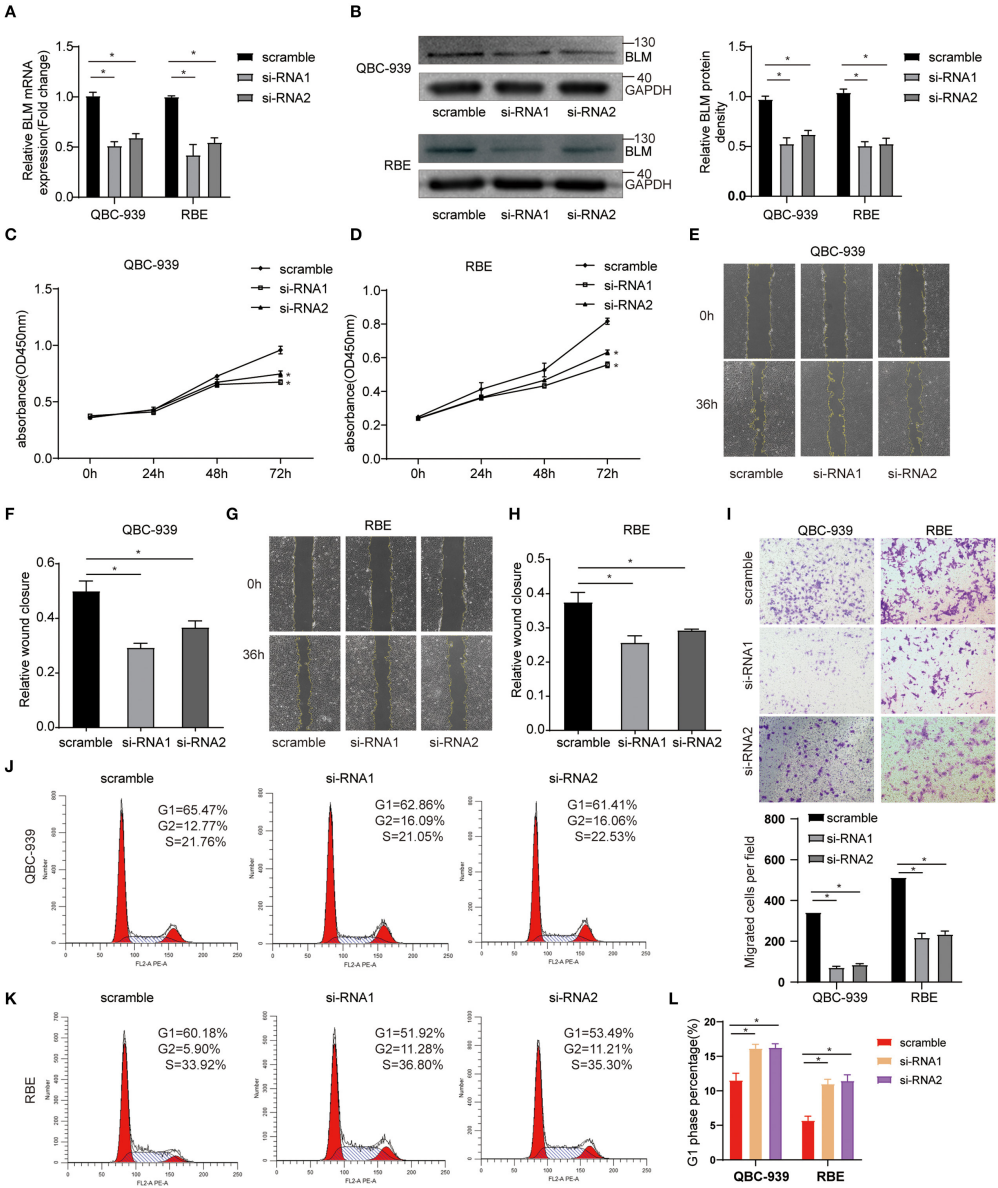
BLM silencing inhibited the proliferation and migration of cholangioca-rcinoma cells and induced cell cycle arrest. **(A)** qRT-PCR validation of BLM knockdown. **(B)** WB validation of BLM knockdown. **(C,D)** CCK8 assay analysis of the proliferation of QBC-939 cells **(C)** and RBE cells **(D)** transfected with BLM small interfering RNA (siRNA) (si-RNA1 and si-RNA2) or control siRNA (scramble). **(E–H)** The effects of BLM knockdown on QBC939 cell and RBE cell migration ability were assessed by wound-healing assay; representative photographs of wound-healing assay **(E,G)**, and quantitative results shown as relative migration rates **(F,H)**. **(I)** The effects of BLM knockdown on QBC939 cell and RBE cell migration were evaluated by Tanswell assay. Representative photographs of the transwell assay (upper panel) and quantitative results shown as number of migrated cells per field (lower panel). **(J)** Flow cytometric analysis of the cell cycle with BLM knockdown and control group in RBE cells. **(K)** Flow cytometric analysis of the cell cycle with BLM knockdown and control group in QBC-939 cells. **(L)** G2-phase analysis of the cell cycle with BLM knockdown and control cells. Data are represented as the mean ± SEM of 3 independent experiments. *p < 0.05 vs. control group.

To study the function of BLM *in vitro*, CCA cell lines (QBC-939 and RBE) were transfected with control siRNA (scramble) or BLM siRNA (si-RNA1 and si-RNA2). *Via* PCR and western blot analysis of the harvested cells, we confirmed that BLM expression was significantly knocked down in BLM siRNA transfected cells than that in control groups at both the mRNA (Figure 8A) and protein (Figure 8B) levels. Using the CCK8 assay, we analyzed the viability of BLM-downregulation cells. Our results demonstrated that the downregulation of BLM expression significantly restrained the proliferation of both cell lines (Figures 8C,D). Next, we also assessed the effect of BLM on CCA cell migration *in vitro*. The downregulation of BLM significantly inhibited the migration of QBC-939 and RBE cells (Figures 8E–I). These results suggest a promotive role of BLM in CCA cell proliferation and migration. Moreover, we checked whether BLM silencing has cell cycle arrest effect in CCA cell lines. Cell cycle analysis performed by flow cytometry revealed that downregulation of BLM increased the cell percentage in G2 phase and decreased those in G1 phase and S phase (Figures 8J–L). It indicates that BLM can promote the CCA progression by modulating the cell cycle.

## Discussion

CCA is a highly malignant and heterogeneous neoplasm with a largely unknown molecular basis (27, 28). Because CCA is highly invasive and has no efficient screening methods, once diagnosed, only approximately one-third of patients are suitable for radical resection. However, even undergoing R0 resection, the disease recurrence rate is still high for patients with CCA, with an informed incidence of 60 and 80% at a median follow-up time of 2 and 5 years, respectively (29–31). For patients with late-stage CCA who have lost the chance of radical operation, the 5-year survival rate of non-surgical treatment is only 5% (28, 32). Therefore, exploring the pathogenesis of CCA and searching for key molecules closely associated with the occurrence, progression, and prognosis of the disease is particularly significant for the current diagnostic and treatment strategies of patients with CCA. In this study, first, we determined the blue module most relevant to the pathological grade of CCA by employing WGCNA. Five candidate genes were identified. After subsequent analyses, a real hub gene tightly related to the progression and prognosis of CCA was screened. Our findings may help to improve the ability of treatment decision-making, risk assessment, and prognosis prediction for patients with CCA.

The biological network can intuitively display the interrelationships between various functional elements in the biological system and provide an important platform for the characteristics of the study objects at the system level (33, 34). In combination with the traditional one-dimensional molecular biology research methods, network analysis methods can more accurately illustrate the characteristics of biological systems (35). Gene co-expression network research, as an essential component of biological network research, can aid in obtaining multiple gene modules based on the gene expression profile by analyzing the expression correlation of multiple genes and in studying the association between genes and external traits at the gene module level (36, 37). We conducted a WGCNA to identify the co-expression modules correlated to the CCA progression. A total of 5,378 filtered DEGs were utilized to build a co-expression network, and 29 modules were screened. Loss of differentiation is a common event in tumor progression, and a high histological grade largely has an intimate correlation with an unfavorable prognosis (38). Here, it was found that the blue module had the highest correlation with tumor grade, which means that blue module genes are closely related to clinical traits. GO analysis can describe how genes act in biological systems and can put these descriptions in a computable format, while a KEGG pathway enrichment analysis displays higher-order functional significance and indicates the value of the cell or the organism from its genome data (39, 40). In line with the published data, the enrichment of blue module genes in several GO terms, such as DNA replication, cell–cell junction, mitotic cell cycle checkpoint, and cadherin binding, confirms their involvement in the development of CCA (41, 42). Moreover, the KEGG pathway enrichment results of the identified module genes also indicate their involvement in CCA pathogenesis. For example, hepatitis C virus (HCV) infection is identified as an important risk factor for the development of CCA (43); recent progress has shown that cellular senescent markers can distinguish cholangiocellular carcinoma from duct adenoma, implying a possible role in the pathophysiology of CCA (44, 45). Additionally, cell cycle changes are particularly important for the growth of malignant tumors (42). According to the GO and KEGG enrichment results, we propose that these blue module genes are closely related to the cell cycle regulation and CCA development.

A study on the cellular function requires a deep understanding of functional interactions between the expressed proteins. The online STRING database can collect and integrate the cellular function data from the investigated and predicted PPI information for a great number of organisms (19). By constructing a PPI network based on the STRING database, we can study the interaction of proteins expressed by a group of genes and identify the key network proteins among them. In addition, hub genes of the co-expression network are characterized as highly related to the clinical traits of CCA samples in TCGA. A total of five candidates (BLM, RPS3, GGH, NUP107, and CCT2) were distinguished, which were common hub genes in both the co-expression network and PPI network of the blue module. All of them were reported to exert important roles in the pathogenesis of some carcinomas (46–50). Here, we carried out an expression pattern analysis and a survival analysis to explore the real hub genes, and BLM with high diagnostic and prognostic value was ultimately identified. Meanwhile, in the cell and tissue levels, we clarified the expression of the real hub gene, and the results further confirmed that BLM possesses completely different expression patterns in CCA compared with the non-tumor control.

The RecQ helicase family, which includes RECQ1, RECQ4, RECQ5, Werner protein (WRN), and BLM, plays an indispensable role in DNA replication, repair, RNA transcription, and telomere maintenance (51, 52). Germ cell mutations in these genes can lead to inherited diseases characterized by premature aging and/or cancer propensity, suggesting a pivotal role of the RecQ family in genome stability (53). For example, WRN, BLM, and RECQ4 are closely associated with the Werner syndrome, Bloom syndrome, and Rothmund Thomson syndrome, respectively, for which there are currently no effective therapies (54). Meanwhile, recent studies have demonstrated that RecQ helicases are also involved in the pathogenesis of multiple human sporadic tumors. RECQ1 may serve as a vital mediator in promoting the non-small cell lung cancer progression *via* the regulation in epithelial to mesenchymal transition (EMT) (55). Lieb et al. proved that the WRN inactivation selectively decreased the viability of microsatellite instability-high (MSI-H) colorectal and endometrial cancer cell lines (56). Moreover, RECQ4 overexpression accelerated the DNA replication rate and reduced chemosensitivity in breast cancer, thus promoting the tumor progression in established breast cancers (57). Patients with Bloom syndrome, who have BLM germ-line mutation resulting in striking decreases in both mRNA and protein expression levels of BLM, are apt to develop diverse malignancies including breast cancer, prostate cancer, and lung cancer (52, 58). Furthermore, previous studies have supposed that BLM serves as a cancer suppressor through targeting the proto-oncogene c-MYC, and recent researches also proposed that a series of malignancies are associated with the overexpression of the MYC gene and loss of BLM function (54, 59). Chandra et al. further confirmed this view in their research, which suggested that BLM indeed promoted the degradation of Myc proto-oncogene protein and subsequently led to an obstruction in the MYC-dependent induction of tumors (60). Interestingly, recent studies have also demonstrated that BLM is highly expressed in multiple cancer tissues and even acts as a novel cancer biomarker. For example, Chen et al. showed that BLM protein could promote cell proliferation and inhibit apoptosis *via* activating AKT signaling and downregulating the PTEN expression in prostate cancer cells (61); Arora et al. *via* analyzing molecular profiling in a large cohort of 1,980 breast cancer samples, found that BLM mRNA overexpression was linked to a poor breast cancer-specific survival and that high cytoplasmic BLM indicated aggressive phenotypes (62). Our current research has revealed that BLM expression is increased in CCA tissues compared with paracancerous samples, which has been further verified in assays *in vitro*, and high BLM expression is correlated with the tumor progression and adverse prognosis. Moreover, we found that BLM silencing represses the CCA cell proliferation and migration *in vitro*. More recently, the dysregulation of DNA methylation patterns has been increasingly perceived as a pivotal cellular procedure during both the initiation and progression of oncogenesis (63, 64). DNA hypomethylation of genome-wide regions, especially CpG islands, is a common epigenetic remodeling in CCA and is in correlation to the activation of some proto-oncogenes and the existence of chromosomal instability (65, 66). By using the online tools DiseaseMeth 2.0 and MEXPRESS, we also investigated the DNA methylation status that possibly resulted in the abnormal expression of BLM in CCA. BLM methylation was reduced in cancer tissues compared with the adjacent normal samples, which was in accordance with the observed upregulation of this real hub gene in CCA. Then, GSEA was executed to explore the possible pathogenesis of BLM in CCA. The results showed that various cell cycle-related KEGG pathways, such as the CELL CYCLE pathway, MISMATCH REPAIR pathway, and DNA REPLICATION pathway, were enriched in the high-expression group of BLM, indicating their contribution to CCA cell proliferation, and *in vitro* experiments results further confirmed the vital role of BLM in regulating the cell cycle of CCA.

## Conclusion

In summary, our present study attempted to explore potential molecular mechanisms in CCA by employing a range of bioinformatics analyses. We identified a real hub gene (BLM) that might cause the progression and poor prognosis of CCA, and BLM might affect these biological processes by regulating the cell cycle process. All these findings contribute to the clinical diagnosis, treatment, and prognostic evaluation of CCA. Next, we will perform experimental research to explore the specific molecular biological mechanisms underlying the involvement of BLM in modulating the CCA occurrence and development.

## Data Availability Statement

The datasets presented in this study can be found in online repositories. The names of the repository/repositories and accession number(s) can be found at: https://www.ncbi.nlm.nih.gov/geo/, GSE76297, GSE132305, https://portal.gdc.cancer.gov/, TCGA-CHOL.

## Ethics Statement

The studies involving human participants were reviewed and approved by Shanghai Chaoshi Biotech Company Ethics Committee Shanghai Outdo Biotech Company, Shanghai, China. The patients/participants provided their written informed consent to participate in this study.

## Author Contributions

ZS conceived the study plan and contributed to the revision of the final manuscript. XD and CZ performed the experiments, analyzed the data, and finished the manuscript writing. CY, WW, XY, DX, and XZ participated in data collection and literature search. QZ contributed to the manuscript writing and data interpretation. ML guided the cell experiments. All authors contributed to the article and approved the submitted version.

## Conflict of Interest

The authors declare that the research was conducted in the absence of any commercial or financial relationships that could be construed as a potential conflict of interest.
